# Multiple Options, One Objective: Clearer Decisions in Resectable Non-Small Cell Lung Cancer

**DOI:** 10.3390/cancers18182963

**Published:** 2026-09-14

**Authors:** Elio Nohra, Tala Najdi, Axelle Serena El Karak, Ghadi Dagher, Fadi El Karak

**Affiliations:** 1 Faculty of Medicine, Saint Joseph University of Beirut, Beirut 1104 2020, Lebanon; elio.nohra@net.usj.edu.lb (E.N.); tala.najdi@net.usj.edu.lb (T.N.); ghadi.dagher@net.usj.edu.lb (G.D.); 2 Department of Hematology-Oncology, Hotel-Dieu de France University Hospital, Beirut 1104 2020, Lebanon; 3 Faculty of Medicine, European University Cyprus, Nicosia 1516, Cyprus; ae231590@students.euc.ac.cy

**Keywords:** non-small cell lung cancer, target therapy, immunotherapy, surgery, adjuvant therapy

## Abstract

Background: Early-stage non-small cell lung cancer (NSCLC) accounts for nearly 50% of cases. Surgery remains the cornerstone of curative treatment, but five-year survival is suboptimal, necessitating adjuvant, neoadjuvant, and perioperative strategies including chemotherapy, targeted therapy, and immunotherapy. Methods: We reviewed randomized trials and conference abstracts from the last decade up to August 2025 on adjuvant, neoadjuvant, and perioperative systemic therapies in resectable NSCLC, focusing on major pathological response, pathological complete response, event-free survival (EFS), and overall survival (OS), and also highlighting several ongoing trials in this setting. Results: The review generated a simplified treatment algorithm to assist clinicians in navigating the multiple therapeutic options for resectable NSCLC. Cisplatin-based adjuvant chemotherapy remains standard for stage II–IIIA disease, while targeted therapy (osimertinib, alectinib) improves DFS in EGFR- and ALK-positive tumors. Adjuvant immunotherapy (atezolizumab, pembrolizumab) shows benefit primarily in PD-L1-positive patients, though regulatory approvals differ depending on PD-L1 expression thresholds. Neoadjuvant nivolumab plus chemotherapy significantly improved EFS and OS, particularly in stage IIIA (CheckMate 816). Perioperative regimens with nivolumab, durvalumab, or pembrolizumab show consistent EFS gains, most pronounced in stage III N2 patients. Emerging biomarkers such as residual viable tumor and circulating tumor DNA after surgery may further personalize therapy. Conclusions: Treatment of resectable NSCLC should be individualized based on stage, nodal status, PD-L1 expression, molecular alterations and pCR. Upfront surgery with adjuvant therapy is appropriate for lower-stage disease (IB–IIA), while perioperative chemoimmunotherapy offers maximal benefit in stage II–III, especially N2-positive patients. Biomarker-driven strategies will continue to refine patient selection to stop or continue postoperative treatment.

## Simple Summary

Treatment options for resectable non-small cell lung cancer have expanded substantially with the introduction of targeted therapies and immune checkpoint inhibitors. This review summarizes current evidence for adjuvant, neoadjuvant, and perioperative systemic treatment and proposes a practical approach to treatment selection. Surgery remains central to curative treatment, particularly for early-stage disease, while perioperative chemoimmunotherapy is increasingly relevant for stage II–III disease, especially in patients with N2 involvement. Molecular alterations such as EGFR mutations and ALK rearrangements guide the use of adjuvant targeted therapy, whereas PD-L1 expression can inform adjuvant immunotherapy decisions. Emerging biomarkers, including pathological response, residual viable tumor, and circulating tumor DNA, may further help individualize treatment but their utility for guiding decisions requires prospective validation.

## 1. Introduction

Early-stage lung cancer represents a significant proportion of cases, with approximately 50% of non-small cell lung cancer (NSCLC) patients presenting with either localized (stages I–II) or locally advanced (stage III) disease [1]. Surgery has traditionally been the cornerstone of treatment in this setting; however, when performed alone, it yields a 5-year overall survival (OS) rate of only about 59% [2], highlighting the need for additional strategies to improve long-term outcomes.

Adjuvant therapies were initially introduced to eradicate micrometastatic disease and reduce recurrence risk [3]. Chemotherapy was the first treatment to demonstrate a modest survival benefit in advanced NSCLC, yielding an absolute improvement of approximately 5% at 5 years [4], followed more recently by the advent of targeted therapies and immune checkpoint inhibitors administered postoperatively. Neoadjuvant approaches subsequently emerged, offering the advantage of earlier treatment of micrometastatic disease, immune system priming, and potential tumor downstaging before surgery. The therapeutic landscape of NSCLC is also evolving with the emergence of antibody–drug conjugates (ADCs) with several ADCs receiving regulatory approval for advanced disease [5,6].

More recently, perioperative strategies, combining pre- and post-operative therapies, have gained prominence, particularly in the era of immunotherapy. These approaches aim to maximize treatment exposure, reduce recurrence risk, and further improve cure rates.

Thus, the management of early-stage lung cancer increasingly requires individualized consideration of adjuvant, neoadjuvant, and perioperative approaches. In this review, we discuss the current options and present an algorithm to guide clinicians in selecting the most appropriate therapeutic strategy.

## 2. Methods

### 2.1. Search Strategy

**Figure 1:**
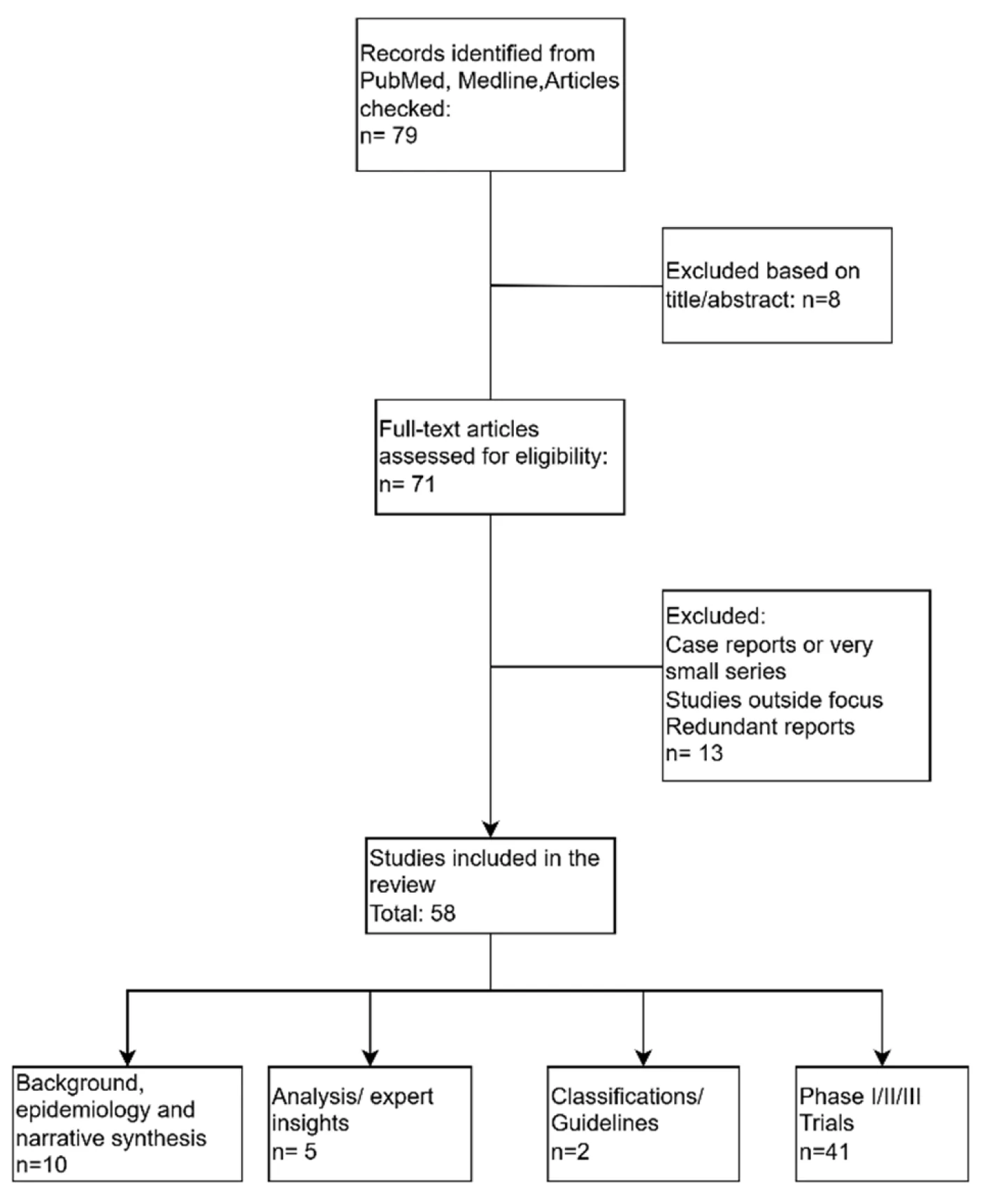
Research process chart.

We performed a comprehensive search of the literature up to August 2025 using the following databases: PubMed, MEDLINE, Cochrane Central Register of Controlled Trials (CENTRAL), and ClinicalTrials.gov. In addition, abstracts from major oncology conferences—including the American Society of Clinical Oncology (ASCO), European Society of Medical Oncology (ESMO), American Association for Cancer Research (AACR), and European Lung Cancer Congress (ELCC) were screened for relevant studies. The detailed search strategies are summarized in the accompanying chart below (Figure 1).

### 2.2. Inclusion and Exclusion Criteria

Studies were included if they enrolled patients with NSCLC confirmed by histological or cytological examination, evaluated neoadjuvant, perioperative, or adjuvant immunotherapy (or directly compared these approaches), and reported at least one relevant outcome, including major pathological response (MPR), pathological complete response (pCR), event-free survival (EFS), and overall survival (OS). Studies were excluded from the results analysis if they were meta-analyses, systematic or narrative reviews, letters, surveys, case reports, book chapters, or were incomplete or lacked sufficient outcome data. Reviews and meta-analyses were used only as background references where appropriate and were not included in the extraction of results.

### 2.3. Data Extraction

Two authors independently extracted data from all eligible studies. Any discrepancies in data extraction or risk of bias assessment were resolved by a third author. The remaining authors reviewed and approved the final data extraction. Extracted data included trial details (trial name, first author, publication source, year, trial phase, and ClinicalTrials.gov identifier), patient demographics and clinical characteristics (sample size, pathological stage, histology, mutation status, and PD-L1 expression when available), and clinical outcomes, including hazard ratios (HRs) with 95% confidence intervals (CIs) for EFS and OS, as well as MPR and pCR rates.

Since most outcomes were reported narratively, the evidence for each treatment approach (neoadjuvant, adjuvant, and perioperative) was synthesized through informal multidisciplinary discussion among the co-authors. Based on this evidence synthesis, a decision algorithm was developed and finalized by consensus among all authors.

## 3. Results

### 3.1. Adjuvant Setting

For patients with resectable NSCLC having >IB tumors (≥4 cm), adjuvant therapy was originally developed to improve survival by reducing recurrence risk. Platinum-based chemotherapy was rigorously evaluated in the IALT and ANITA trials. In IALT, patients assigned to adjuvant chemotherapy demonstrated significantly higher five-year survival compared with observation (44.5% vs. 40.4%; HR 0.86) [7]. Similarly, ANITA reported an eight-year OS improvement of 8.4–8.6% at five and seven years, respectively [8]. Although these gains represent a modest absolute survival benefit of approximately 5% at five years, cisplatin-based chemotherapy remains the default regimen for medically eligible patients.

The landscape of adjuvant therapy encompassing stage IB tumors through stage IIIA and selected stage IIIB disease, as defined by the 7th and 8th edition of the AJCC, has evolved from a uniform chemotherapy approach to a personalized strategy that integrates immunotherapy and targeted therapy. Atezolizumab was the first FDA-approved adjuvant immunotherapy for patients with completely resected PD-L1 ≥ 1% NSCLC, demonstrating clinically meaningful benefit. The IMpower010 trial enrolled 1280 patients with resected stage IB tumors ≥ 4 cm through stage IIIA NSCLC, all of whom had received prior cisplatin-based adjuvant chemotherapy. Following recovery, participants were randomized to receive one year of atezolizumab or best supportive care. In patients with stage II–IIIA disease and PD-L1 expression ≥1%, atezolizumab significantly improved disease-free survival (DFS; HR 0.66 [95% CI 0.50–0.88; *p* = 0.0039]). The greatest benefit was observed in the PD-L1 TC ≥ 50% subgroup (DFS not estimable vs. 41.1 months; HR 0.48), regardless of EGFR or ALK mutation status. Five-year updates confirmed the durability of this DFS advantage and suggested a trend toward improved OS [9]. The European Medicines Agency (EMA) restricted atezolizumab approval to PD-L1 ≥ 50%, unlike the FDA, which approved PD-L1 ≥ 1% [10,11].

Pembrolizumab further expands the adjuvant immunotherapy landscape by offering benefit across NSCLC populations without mandatory PD-L1 testing. In the KEYNOTE-091/PEARLS trial, 1177 patients with resected stage IB ≥ 4 cm through stage IIIA disease were randomized after platinum-based chemotherapy to receive one year of pembrolizumab or placebo. The trial showed improved DFS in the intention-to-treat population ITT (median DFS 53.6 months vs. 42.0 months HR 0.76.) Interestingly, in patients with PD-L1 TPS ≥ 50%, no significant DFS difference was observed (HR 0.82), highlighting asymmetrical outcomes in exploratory subgroups. OS as a secondary endpoint was not different between both arms (HR = 0.87) [12]. Nevertheless, the overall hazard ratio of DFS supported regulatory approval; pembrolizumab is FDA-approved and NCCN-recommended as adjuvant therapy for stage IB–IIIA NSCLC regardless of PD-L1 status, though benefit in PD-L1 negative patients is less certain.

By introducing a biomarker-driven framework, adjuvant targeted therapy has dramatically changed the management of early-stage EGFR-mutated NSCLC. The ADAURA trial enrolled 682 patients with resected stage IB through IIIA EGFR-positive tumors, randomizing them to three years of osimertinib or placebo, independent of prior chemotherapy. Osimertinib demonstrated robust DFS benefit at 24 months (90% vs. 44%; HR 0.17) and significant OS advantage (89% vs. 52%; HR 0.20), establishing it as the preferred adjuvant intervention for this molecular subset [13]. Earlier studies with first-generation EGFR TKIs, such as gefitinib in CTONG1104, showed DFS improvement but failed to translate into overall survival benefit [14]. Consequently, osimertinib alone is now recognized as the standard adjuvant therapy for patients with sensitizing EGFR mutations. Additionally, in 2024, the ALINA trial established alectinib as a new adjuvant standard for up to 24 months in ALK-positive resectable NSCLC, showing a 2-year DFS of 93.8% vs. 63% with chemotherapy (HR 0.24) [15].

Several other adjuvant immunotherapy trials in resected NSCLC are still forthcoming. The BR31 trial (durvalumab, *n* = 1360) did not demonstrate significant improvements in DFS or OS, contrasting with earlier successes of immunotherapy [16]. The ANVIL trial (nivolumab, *n* = 903) is ongoing, with DFS and OS results pending [17]. The ALCHEMIST (Adjuvant Lung Cancer Enrichment Marker Identification and Sequencing Trials) program is a set of ongoing randomized clinical trials in patients with completely resected early-stage NSCLC to evaluate whether adjuvant immunotherapy such as in the ACCIO trial (e.g., pembrolizumab) or targeted therapy (e.g., for EGFR/ALK alterations) after standard chemotherapy can improve long-term outcomes such as DFS and OS [18]. These trials are summarized in Table 1 below.

### 3.2. Neoadjuvant Setting

Between 2018 and 2022, several Phase II trials, including Forde et al. and NeoSTAR for nivolumab, Shu et al. and LCM3 for atezolizumab established the safety of immunotherapy in the neoadjuvant setting [19,20,21,22] as shown in Table 2. In 2022, immunotherapy formally expanded from the adjuvant to the neoadjuvant setting, with nivolumab becoming the first agent approved in combination with platinum-doublet chemotherapy for resectable stage IB (≥4 cm)–IIIA NSCLC showing a better EFS (median 31.6 vs. 20.8 months; HR 0.63) regardless of PDL1 expression. Surgical cancellation occurred in 15.6% of patients in the nivolumab-plus-chemotherapy arm (12% in stage IB–II, 17% in stage IIIA) [23]. At the 4-year update of CheckMate 816, nivolumab plus chemotherapy significantly improved EFS particularly in stage IIIA patients, compared with chemotherapy alone (median 43.8 vs. 18.4 months; HR 0.66) and was associated with higher circulating tumor DNA clearance rates (56% vs. 35%). The benefit was consistent across surgical subgroups, and a sustained OS advantage was observed (HR 0.71; +13% at 4 years), although the interim analysis did not reach statistical significance. To date no other immunotherapy agent has been approved based on a Phase III trial conducted exclusively in the neoadjuvant setting [24].

### 3.3. Perioperative Setting

In contrast, all three immunotherapy agents, nivolumab, durvalumab, and pembrolizumab have received regulatory approval from both the FDA and the EMA for use in the perioperative treatment of resectable stage IIA–IIIB NSCLC between 2023 and 2024.

The NADIM I and II trials were single-arm, Phase II studies investigating perioperative nivolumab, demonstrating manageable safety and providing the foundation for subsequent Phase III trials [25,26]. CheckMate-77T (NCT04025879) then enrolled 461 patients with stage IIA to IIIB and compared perioperative nivolumab (3 neoadjuvant doses plus 12 adjuvant doses) plus platinum-doublet chemotherapy versus chemotherapy plus placebo. The trial showed an 18-months 70.2% versus 50.0% EFS (HR 0.58) [27]. KEYNOTE-671 (NCT03425643) enrolled 797 patients with resectable stage II–IIIB NSCLC who received neoadjuvant pembrolizumab for 4 cycles in combination with platinum-based chemotherapy, followed by surgery and adjuvant pembrolizumab for up to 13 cycles, showing a 36.6 months EFS of 47.2% versus 18.3% (HR 0.59) and OS NR versus 52.4% (HR = 0.72) [28]. Following the demonstration of safety in Phase II SAKK 16/14, perioperative durvalumab in stage IIIA was further evaluated in the AEGEAN trial [29]. This trial (NCT03800134) included 802 patients with IIA to IIIB (N2) treated with durvalumab plus chemotherapy for 4 cycles followed by adjuvant durvalumab for 12 cycles versus chemotherapy plus placebo and demonstrated EFS of 73.4% versus 64.5% (HR 0.68) and pCR of 17.2% versus 4.3% [30].

All three trials, CheckMate-77T, AEGEAN, and KEYNOTE-671, used the 8th edition of the AJCC staging system. In CheckMate-77T, patients with EGFR mutations or ALK rearrangements were excluded, in AEGEAN, 7.7% of patients presented with ALK or EGFR alterations but were excluded from ITT analysis, while in KEYNOTE-671, 4.1% of participants harbored EGFR mutations but there was not enough evidence to recommend it over targeted therapy for this group and all three trials included patients regardless of PD-L1 expression levels.

The NEOTORCH trial builds upon the current standard of neoadjuvant chemoimmunotherapy, highlighting perioperative immunotherapy in stage III with toripalimab as a potential new paradigm; however, this agent is not FDA-approved at present [31]. Numerous ongoing trials are currently investigating the addition of perioperative targeted therapy, including Geometry-N for MET, LIBRETTO-432 for RET, NAUTIKA for BRAF V600E, KRAS G12C, NTRK, RET, and ROS1, as well as LUN-109 and NeoCOAST-2 (perioperative combination with immunotherapy for all alterations except EGFR+/ALK+) [32,33,34,35,36].

### 3.4. Investigational Role of ADCs in Early-Stage NSCLC

Antibody-drug conjugates (ADCs) represent a logical extension of the “targeted delivery” paradigm into earlier lines of NSCLC therapy, distinct in mechanism from ICIs: they pair a tumor–antigen-targeting antibody with a cytotoxic payload via a cleavable linker, enabling selective intracellular drug release and, through the bystander effect, the killing of antigen-low neighboring cells independent of T-cell priming. This extension into the perioperative setting builds on the efficacy already shown in metastatic disease and is now being tested across three complementary trials. In the adjuvant space, TROPION-Lung12 (NCT06564844) randomizes patients with resected, ctDNA-positive or high-risk pathology stage I adenocarcinoma NSCLC to datopotamab deruxtecan (Dato-DXd) plus the PD-1/TIGIT bispecific rilvegostomig, rilvegostomig alone, or observation/chemotherapy, using DFS as the primary endpoint and ctDNA rather than PD-L1 as the enrichment biomarker [37]. In the neoadjuvant setting, the Phase II platform trial NeoCOAST-2 paired Dato-DXd with single-agent platinum chemotherapy and durvalumab in resectable stage IIA–IIIB NSCLC, achieving the highest pCR among its experimental arms (35.2%), early evidence that an ADC-containing regimen can outperform conventional ICI-chemotherapy doublets pathologically [38]. A parallel strategy targets HER2-altered disease: the Phase II HERCULES trial (NCT07428044) is evaluating trastuzumab deruxtecan (T-DXd), an ADC already established in later-line metastatic HER2-mutant/overexpressing NSCLC, in the neoadjuvant setting [39]. None of these trials has yet reported mature DFS, EFS or OS data, so this evidence should be framed as investigative.

## 4. Discussion

### 4.1. Mechanisms and Clinical Impact of Adjuvant ICIs

ICIs restore anti-tumor T-cell activity by blocking inhibitory checkpoint interactions. Anti-PD-1 (nivolumab, pembrolizumab) and anti-PD-L1 (atezolizumab, durvalumab) agents disrupt the PD-1/PD-L1 axis that tumor cells exploit to evade cytotoxic T-lymphocyte killing, while anti-CTLA-4 agents act earlier, at the priming phase in lymph nodes, by blocking CTLA-4-mediated downregulation of T-cell activation [40]. ICIs’ benefit differs by treatment line, each mapping to a distinct immunological objective. In unresectable stage III disease, ICIs provide immune consolidation after chemoradiotherapy [41]. In the adjuvant setting, the rationale is distinct from metastatic disease: ICIs are hypothesized to eliminate minimal residual disease and micrometastases left after complete resection; by sustaining an activated, memory-competent T-cell pool capable of immunosurveillance, a mechanistic shift from response-driven to eradication-driven immune activity underlies why DFS rather than ORR is the primary endpoint in resected NSCLC trials, and why PD-L1 expression behaves less predictably as a biomarker in this context than in advanced disease.

### 4.2. Neoadjuvant vs. Surgery Followed by Adjuvant Immunotherapy

Adjuvant therapy, however, does not exploit the opportunity for immune priming in the presence of the intact tumor, since it is administered only after the primary lesion and its antigenic burden has already been surgically removed. Adjuvant therapy has historically aimed to eradicate micrometastatic disease after surgery, but it does not exploit the opportunity for immune priming in the presence of the intact tumor. This limitation inspired the use of neoadjuvant immunotherapy, such as nivolumab, administered before resection. By treating the tumor and regional lymph nodes in situ, neoadjuvant immunotherapy stimulates stronger and broader systemic immune responses, as shown by immune profiling studies [42]. However, the immunological effects of neoadjuvant therapy extend beyond the PD-1/PD-L1 axis, as the tumor microenvironment contains multiple immunosuppressive mechanisms that may influence treatment response. Importantly, radiological response, pathological response, and ctDNA dynamics reflect different aspects of tumor biology and should therefore be interpreted as complementary rather than interchangeable measures of treatment response. Additional advantages include tumor downstaging, improved operability, and enhanced tolerability in the preoperative setting [43].

Because surgery remains the cornerstone of curative treatment, it is essential in both neoadjuvant and perioperative settings to consider the risk of treatment-related surgical delays or cancellations; patients may not undergo surgery for a variety of reasons so careful patient selection and multidisciplinary assessment are therefore essential, particularly for patients considered at higher risk of not proceeding to surgery, in whom upfront surgery may be preferable. Although these approaches generally do not preclude surgery, treatment-related changes may result in hilar fibrosis, nodal response, altered tissue planes, conversion to open surgery, changes in the extent of pulmonary resection, and potentially increased postoperative morbidity [44]. Therefore, the proportion of patients who ultimately undergo surgery, R0 resection rates, and postoperative complications are important measures to consider. Surgical delays and cancellations should also be reported separately in the trials where possible, as combining these two distinct outcomes into a single percentage makes it difficult to interpret the actual surgical feasibility of a given treatment strategy.

In stage IB–IIA disease, the risk of occult systemic disease is relatively low compared with more advanced stages. In this setting, avoiding delays is particularly important to ensure that potentially curative surgery is not missed. Upfront surgery is therefore generally preferred, especially given the low risk of R1 resection (~4%) [45]. This approach can be followed by adjuvant therapy, including immunotherapy when indicated. Outcomes in these early stages remain favorable, with 5-year OS of 57.3% in stage IB, and 3-year recurrence-free survival of 74% in stage IB and 58% in stage II [46,47], supporting immediate surgical management in this population.

In contrast, later stage NSCLC (stage IIB, IIIA–IIIB) is a heterogeneous group with a historically poorer prognosis, with 5-year OS ranging from 24–41%. In this context, effective control of micrometastatic disease becomes particularly important. The risk of incomplete resection is also higher; NCDB analyses report R1 resection rates of more than 10% [40], so the lack of robust in situ immune priming in untreated disease may limit long-term outcomes. Neoadjuvant immunotherapy, including nivolumab, generally does not compromise surgical feasibility and may improve systemic disease control, supporting its use in this setting [48].

### 4.3. Which Adjuvant Therapy to Choose?

In resected stage IB and IIIA NSCLC, adjuvant chemotherapy provides a modest absolute survival benefit of approximately 5% at five years as shown in the LACE analysis [49]. Despite this limited gain, cisplatin-based doublets remain the standard of care serving the chemotherapy backbone for subsequent post-resection immunotherapy when indicated. However, osimertinib for EGFR-mutant and alectinib for ALK-positive disease both showed superior efficacy as adjuvant monotherapy, regardless of prior chemotherapy. Although adjuvant chemotherapy was not mandatory, 60% of patients in ADAURA and 84% in ALINA received it prior to randomization [13,15]. Immunotherapy, however, is generally contraindicated in this setting. Safety data show that combining checkpoint inhibitors with EGFR tyrosine kinase inhibitors significantly increases the risk of immune-mediated toxicities, including pneumonitis and hepatitis, without providing additional efficacy in the adjuvant setting [50]. Furthermore, high PD-L1 expression (≥50%) in EGFR-mutant NSCLC is associated with worse PFS and OS, serving as a marker of relative resistance to osimertinib. Therefore, the recommended approach for EGFR-positive resected NSCLC is three years of osimertinib following surgery [51].

For patients with resected stage II and IIIA NSCLC that is EGFR- and ALK-negative, adjuvant immunotherapy decisions are guided by PD-L1 expression. Tumors with PD-L1 ≥ 1% may benefit from atezolizumab, with greater PD-L1 expression correlating with larger improvements in DFS. However, this does not automatically translate to using pembrolizumab in the adjuvant setting as it is generally not chosen for patients with PD-L1 > 50%. The benefit use of pembrolizumab is primarily observed in PD-L1-negative or unknown tumors where atezolizumab lacks approval [52].

High PD-L1 expression alone does not always dictate the choice of immunotherapy; disease stage and trial representation must also be considered, as stage IB patients were not included in the ITT in IMpower010 (atezolizumab) but were included in the KEYNOTE trial (pembrolizumab) as shown in the table below (Table 3). Moreover, given the higher representation of stage III patients in IMpower010, atezolizumab is often preferred in stage III if PDL1+. On the other hand, safety profiles should guide patient selection; immune-related adverse events (AEs) are consistent with expectations for checkpoint inhibitors, with grade 3 or higher toxicities observed in approximately 34% of patients receiving pembrolizumab and 22% receiving atezolizumab, while discontinuation rates remained below 20% in both trials. However, these rates should not be interpreted as evidence that one checkpoint inhibitor is intrinsically safer than another, as differences in trial populations, AE definitions, follow-up duration, and reporting may influence the observed rates. The table below (Table 4) provides a comparison of the AEs between the two trials.

While KEYNOTE-091 breaks down immune-mediated adverse events by specific organ system, reporting any-grade pneumonitis (7%), hypothyroidism (22%), and hyperthyroidism (11%) individually, IMpower010 reports immune-mediated toxicity primarily as a composite figure (52% any grade, 8% grade 3–4), making comparison difficult.

Overall, the therapeutic strategy for resected NSCLC should integrate both the molecular landscape and disease stage. Cisplatin-based chemotherapy remains an important option and may serve as the foundation of therapy in selected patients. EGFR-positive tumors are best managed with three years of osimertinib, while ALK-positive tumors receive two years of alectinib, with immunotherapy generally excluded. For ALK-negative, EGFR-wild-type tumors, atezolizumab may be considered in patients with PD-L1 ≥ 50% regardless of stage (II or III). In stage III disease, it may also be considered when PD-L1 is <50%, while pembrolizumab is appropriate for PD-L1-negative or unknown tumors, particularly in stage IB ≥ 4 cm and stage II disease, with a more limited role in stage III.

Circulating tumor DNA (ctDNA) is emerging as a key tool for detecting molecular residual disease (MRD) and predicting recurrence risk, with studies such as BR31 and ALCHEMIST showing that post-resection ctDNA positivity is associated with significantly worse DFS and OS [16,18]. While ctDNA status is increasingly recognized as a prognostic biomarker, its predictive utility has not yet been established. Clinically, ctDNA-guided MRD detection could personalize adjuvant therapy, escalating treatment for MRD-positive patients and de-escalating or omitting adjuvant therapy for ctDNA-negative patients post resection. Such strategies remain investigational and require prospective validation before they can be incorporated into routine clinical practice.

### 4.4. Perioperative vs. Neoadjuvant Approaches

The perioperative setting combines both, offering immune continuity: priming before surgery followed by surveillance after. Real-world studies have questioned the necessity of adjuvant immunotherapy after neoadjuvant treatment, suggesting that its impact may be limited and difficult to interpret, with outcomes often confounded by selection bias [53]. An additional practical point is that in patients where a shorter exposure to immunotherapy is preferred, such as those with pre-existing autoimmune disease, neoadjuvant therapy may be favored, since it requires only three cycles rather than extended perioperative treatment.

In the absence of head-to-head trials, exploratory analyses using propensity score weighting have been conducted, and a patient-level meta-analysis is ongoing. To date, perioperative nivolumab has shown greater EFS benefit, with median follow-ups of 26.5 months in CheckMate 816 and 33.3 months in CheckMate 77T. While neoadjuvant therapy provides immune priming, facilitates surgical resection, and helps reduce recurrence risk, omitting postoperative systemic treatment may allow residual disease to persist, maintaining the risk of relapse [54]. However, these trials cannot be directly compared, and the specific contribution of postoperative nivolumab remains uncertain, particularly in patients achieving pCR or major pathological response after R0 resection. The optimal role and duration of postoperative immunotherapy therefore remain under investigation. Residual viable tumor (%RVT) is a recently investigated prognostic marker in patients receiving neoadjuvant immunotherapy [55]. In the CheckMate 77T analysis, the HR for EFS varied according to pCR, with patients achieving pCR showing a markedly greater benefit (HR 0.32) compared to those without pCR (HR 0.73) [27]. A practical way to assess this is through ctDNA clearance; patients in the chemoimmunotherapy arm who do not achieve pCR and or remain ctDNA-positive are more likely to have residual micrometastatic disease and could derive additional benefit from continuing postoperative treatment, enabling a more individualized treatment approach for resectable disease [56].

The Phase III perioperative trials, including AEGEAN, CheckMate 77T, and KEYNOTE-671, have consistently shown absolute gains in EFS with combined pre- and post-operative immunotherapy. Selected outcomes from neoadjuvant and perioperative trials are shown in the table below (Table 5). Data indicate that stage III NSCLC patients benefit the most, supporting perioperative immunotherapy as an optimal approach to reduce recurrence and improve cure rates in this high-risk population. Specifically, in stage III disease, the AEGEAN reported a more pronounced EFS benefit in N2 patients (HR 0.46) compared to non-N2 patients (HR 0.6), reflecting a greater relative risk reduction in those with higher nodal burden. Moreover, in the recent 9th edition of TNM classification, Stage IIA now includes T1N1 disease, which was previously classified as IIB. This reclassification shifts IIA from a lower-risk, node-negative group to one that often includes nodal involvement, thereby broadening the pool of patients who may benefit from perioperative systemic therapy [57].

Another key consideration in practical patient selection is PD-L1 expression. In perioperative trials, immunotherapy was effective regardless of pathological response or PD-L1 status, which was approximately 50–60% across the three studies, and regulatory approvals have reflected this inclusive approach. When patients show limited response during the neoadjuvant phase, particularly those with low or absent PD-L1, the rationale for continuing adjuvant immunotherapy is unclear. Interim patient data level analysis shows a clear separation of event-free survival curves in PD-L1 < 1% patients with perioperative nivolumab (HR 0.51 [0.28–0.93]), whereas in PD-L1 ≥ 1% patients the curves are close (HR 0.86 [0.44–1.70]), suggesting no significant additional benefit from the adjuvant component [58]. Despite this, trials allowed continuation, and it is still administered in practice, highlighting the gap between trial design, biological rationale, and individualized patient decision-making. Exploratory analyses suggest that higher-risk patients, such as those with stage III N2 disease who are PD-L1 negative or fail to achieve pCR, may benefit more from a full perioperative approach, and ongoing studies are evaluating the contribution of each treatment component.

### 4.5. The Choice of Perioperative Regimen

When selecting a perioperative regimen, all three agents combined with chemotherapy show comparable grade ≥ TRAEs events in approximately 40–45% of patients, primarily driven by chemotherapy. The choice is not fundamentally influenced by AEs, nor by PD-L1 status, since benefits were observed across most subgroups. Still, post hoc analyses from trials such as KEYNOTE-671 showed minimal effect in certain populations (patients >65 years, East Asians, never smokers, or PD-L1 < 1%), with outcomes clustering near the line of no significance. Regulatory discrepancies remain, as nivolumab use in Europe is restricted to PD-L1 positive tumors, while broader approvals exist elsewhere. Beyond these considerations, a practical distinction is that nivolumab is the only checkpoint inhibitor with supporting neoadjuvant-only data, allowing its selection as the perioperative agent while retaining the flexibility to stop or continue after surgery based on individual response. As a result, we prefer nivolumab in this setting.

### 4.6. The Guided Treatment Approach to Adopt

**Figure 2:**
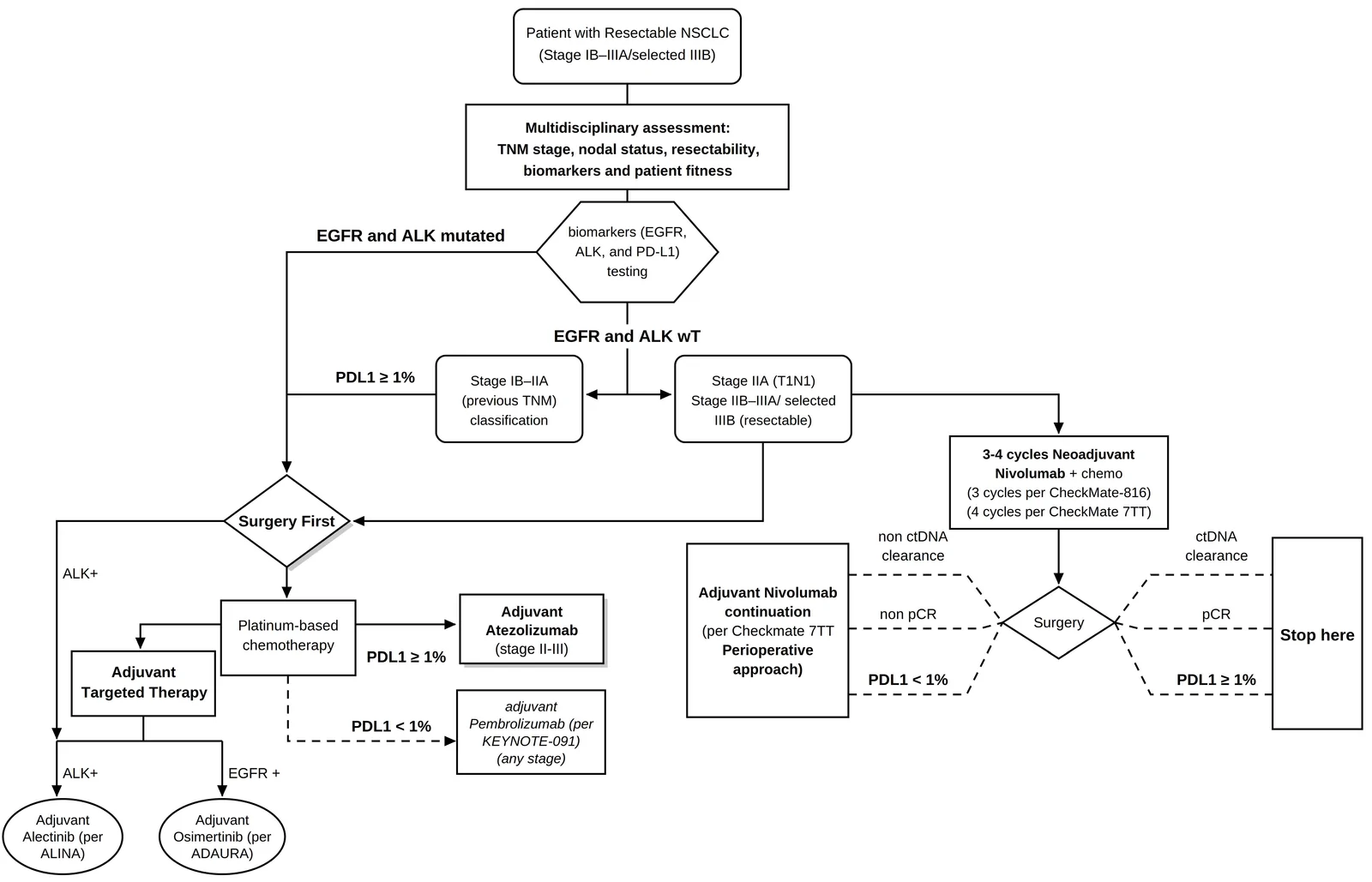
Proposed treatment algorithm for resectable non-small cell lung cancer. Dashed lines indicate potential investigational approaches under evaluation and should not be interpreted as established treatment recommendations.

Based on the results of the reviewed trials and available evidence, we propose the treatment algorithm outlined below (Figure 2).

The initial step should include comprehensive biomarker assessment using NGS for genomic alterations (e.g., EGFR mutations, ALK rearrangements) and immunohistochemistry (IHC) for PD-L1 expression. In patients harboring EGFR or ALK alterations, upfront surgery should be prioritized, followed by appropriate adjuvant targeted therapy, given the substantial benefit observed in this population and the lack of evidence supporting the use of immunotherapy in this setting.

For patients without actionable alterations, treatment decisions should be guided by PD-L1 expression. In tumors with PD-L1 ≥ 50%, adjuvant immunotherapy benefit prompts us to do surgery first. For PD-L1 ≥ 1%, we benefit from surgery followed by adjuvant atezolizumab, based on the available survival data. At present, we do not routinely recommend adjuvant pembrolizumab in this setting due to the absence of mature OS benefit; however, this may evolve pending future results [12]. For earlier stages (IB–IIA disease), upfront surgery remains the preferred approach, given the favorable survival outcomes. Avoiding loss of surgical opportunity in this population is a key consideration in our decision-making, as with the neoadjuvant/perioperative approach approximately 15–24% of patients may not proceed to surgery following neoadjuvant therapy [48].

In stage IIB–IIIA and selected IIIB disease, a neoadjuvant approach with 3–4 cycles of nivolumab plus chemotherapy represents a valid alternative. In patients receiving neoadjuvant therapy, discontinuing immunotherapy after pCR in PD-L1 ≥ 1% has been exploratory analyzed suggesting favorable EFS, but whether postoperative immunotherapy can safely be omitted remains an investigational question [58]. In this context, circulating tumor DNA (ctDNA), when available, may provide additional prognostic information to inform future treatment strategies. Patients achieving ctDNA clearance could potentially be considered for treatment de-escalation, including omission of adjuvant therapy after surgery, although this approach remains investigational. Conversely, in the absence of pCR or persistent disease, a perioperative strategy including adjuvant immunotherapy may be considered according to the available clinical evidence.

#### Limitations in the Use of Biomarkers for Treatment Selection

A key limitation in our proposed algorithm is that pCR, MPR/%RVT, ctDNA, and PD-L1 reflect distinct aspects of tumor biology. PD-L1 represents a pretreatment characteristic of the tumor–immune microenvironment rather than a marker response. While pCR and MPR are established pathological endpoints, ctDNA-MRD is currently prognostic, and %RVT values are tissue-based pathological measures assessed on the resected specimen that remains investigational. Accordingly, pCR and ctDNA status may be discordant, with some patients achieving pCR while remaining ctDNA-positive and vice versa. Therefore, although our proposed algorithm incorporates these markers as potential tools for future treatment personalization, they should not currently be considered equivalent or used alone to justify a treatment option. Prospective validation is required before such biomarker-guided strategies can enter routine clinical practice.

## 5. Conclusions

The treatment landscape for resectable NSCLC has significantly evolved with the integration of immunotherapy; however, surgery remains the cornerstone for achieving a cure. For stage IB–IIA disease, upfront resection is preferred, followed by adjuvant therapy tailored to PD-L1 and molecular status. In cases of stage II–III, especially N2-positive disease, perioperative chemoimmunotherapy offers substantial benefits, combining neoadjuvant immune priming with postoperative consolidation to minimize recurrence. Nodal burden and staging are crucial determinants of outcomes, while PD-L1 status plays a pivotal role in adjuvant therapy selection rather than the perioperative approach. Patients who do not achieve a pathologic complete response (pCR) or have PD-L1-negative tumors may be considered for a comprehensive perioperative strategy, although the optimal approach in these settings remains to be established. Additionally, emerging biomarkers like residual viable tumor and ctDNA clearance post-surgery enhance the ability to personalize adjuvant therapy, thus promoting a more individualized approach to perioperative care. However, these biomarkers reflect distinct aspects of tumor biology, and their potential role in treatment selection, and this requires prospective validation.

## Figures and Tables

**Table 1:** Comparative summary of BR31, ANVIL, ALCHEMIST Chemo-IO trials.

Trial	Stages	Agent/Regimen	Prior Therapy Policy	Primary Endpoints and Outcomes
BR31 [16]	IB (≥4 cm)–IIIA	Durvalumab 1500 mg IV q4w, up to 1 year	Surgery ± adjuvant chemotherapy allowed	No DFS improvement
ANVIL [17]	IB (≥4 cm)–IIIA	Nivolumab 240 mg IV q2w → later q4w, for 1 year	Surgery ± adjuvant chemotherapy or radiotherapy allowed	DFS and OS pending
ALCHEMIST Chemo-IO [18]	IB (≥4 cm)–IIIA	Platinum doublet chemotherapy ×4 cycles → pembrolizumab 200 mg IV q3w for 17 cycles	No prior neoadjuvant or adjuvant systemic therapy	DFS and OS pending

Abbreviations: DFS: disease free survival; OS: overall survival.

**Table 2:** Non-FDA approved neoadjuvant immunotherapy trials in resectable NSCLC.

Trial	Phase	Neoadjuvant Arm	Primary Endpoint	Key Results
Forde et al. [20]	Pilot, Phase I	Nivolumab × 2	Safety, Feasibility	Safe
NeoSTAR [21]	Phase II (multi-arm)	Nivolumab + chemo +/− Ipilimumab × 1	mPR	mPR Nivolumab + Ipilimumab: 38% mPR Nivolumab: 22%
Shu et al. [22]	Phase II (single arm)	Atezolizumab + chemo × 4	mPR	mPR: 50%
LCMC3 [19]	Phase II (single arm)	Atezolizumab × 2	mPR	MPR: 20%

Abbreviations: mPR: major pathological response.

**Table 3:** Stage representation in KEYNOTE-091 (PEARLS) and IMpower010 trials.

Stage	IMpower010	KEYNOTE-091
Stage IB	0	~14.2%
Stage IIA	~34%	~14.2%
Stage IIB	~19%	~14.2%
Stage IIIA	~47%	~30.0%
Stage II (IB–IIIA)	~53%	~55.8%

**Table 4:** Comparison of adverse event percentages between KEYNOTE-091 (PEARLS) and IMpower010 trials.

AE Category	Pembrolizumab (*N* = 580)	Placebo (*N* = 581)	Atezolizumab (*n* = 495)	BSC (*n* = 495)
Any AE	556 (95.9%)	529 (91.0%)	92.5%	70.9%
Grade 3–5 AE	198 (34.1%)	150 (25.8%)	22.0%	11.5%
AE Led to Death	11 (1.9%)	6 (1.0%)	1.8%	0.6%
Treatment-related AE	4 (0.7%)	0 (0%)	67.9%	0%
Serious AE	142 (24.5%)	90 (15.5%)	17.8%	8.5%
Treatment-related serious AE	—	—	7.5%	0%
AE Leading to Treatment Discontinuation	115 (19.8%)	34 (5.9%)	18.2%	0%
AE Leading to Dose Interruption	221 (38.1%)	145 (25.0%)	28.7%	0%

**Table 5:** Oncologic outcomes and surgery cancellation rates in neoadjuvant and perioperative non-small cell lung cancer trials.

Trial	Event-Free Survival (mo)	Overall Survival (%)	pCR (%)	MPR (%)	Grade ≥ 3 TRAEs (%)	Surgery Cancellation After Neoadjuvant Chemoimmunotherapy	Stage III PD-L1
CheckMate 77T (*n* = 461) [27]	18 mo 70.2 vs. 50 (HR = 0.58)	30 mo 78 vs. 72 (HR = 0.85) (ASCO 2025)	25.3 vs. 4.7	35.4 vs. 12.1	32.5 vs. 25.2	22%	64% Stage III 60% PD-L1
KEYNOTE-671 (*n* = 797) [28]	36.6 mo 47.2 vs. 18.3 (HR = 0.59)	71 vs. 64 (HR = 0.72)	18.1 vs. 4	30.2 vs. 11	45 vs. 38	17.9%	70% Stage III 64% PD-L1
AEGEAN (*n* = 802) [30]	12 mo 73.4 vs. 64.5 (HR = 0.68)	Median OS not reached; (HR = 0.89)	17.2 vs. 4.3	33.3 vs. 12.3	42.4 vs. 43.3	22.5% in primarily due to logistical reasons such as scheduling issues (12.3%)	71% Stage III 67% PD-L1
CheckMate 816 (*n* = 358) [24]	21 mo 31 vs. 20.8 (HR = 0.63)	4-y 95 vs. 63 (HR = 0.08)	24 vs. 2.2	36.9 vs. 8.9	33.5 vs. 36.9	15.6%	64% Stage III 51% PD-L1

Abbreviations—EFS: event-free survival; OS: overall survival; MPR: major pathologic response; vs.: versus; HR: hazard ratio; mo: months; y: years; TRAEs: treatment-related adverse events.

## Data Availability

No new data were created or analyzed in this study. Data sharing is not applicable to this article.

## References

[1] Siegel R.L., Miller K.D., Fuchs H.E., Jemal A. (2022). Cancer statistics, 2022. CA Cancer J. Clin..

[2] Garinet S., Wang P., Mansuet-Lupo A., Fournel L., Wislez M., Blons H. (2022). Updated Prognostic Factors in Localized NSCLC. Cancers.

[3] Tang W.-F., Ye H.-Y., Tang X., Su J.-W., Xu K.-M., Zhong W.-Z., Liang Y. (2023). Adjuvant immunotherapy in early-stage resectable non–small cell lung cancer: A new milestone. Front. Oncol..

[4] (1995). Chemotherapy in non-small cell lung cancer: A meta-analysis using updated data on individual patients from 52 randomised clinical trials. Non-small Cell Lung Cancer Collaborative Group. BMJ.

[5] Soni S., Megha K., Shah V.B., Shah A.C., Bhatt S., Merja M., Khadela A. (2025). Unlocking the therapeutic potential of antibody–drug conjugates in targeting molecular biomarkers in non-small cell lung cancer. J. Egypt. Natl. Cancer Inst..

[6] Khadela A., Megha K., Shah V.B., Soni S., Shah A.C., Mistry H., Bhatt S., Merja M. (2024). Exploring the Potential of Antibody-Drug Conjugates in Targeting Non-small Cell Lung Cancer Biomarkers. Clin. Med. Insights Oncol..

[7] Arriagada R., Bergman B., Dunant A., Le Chevalier T., Pignon J.-P., Vansteenkiste J. (2004). International Adjuvant Lung Cancer Trial Collaborative Group. Cisplatin-based adjuvant chemotherapy in patients with completely resected non-small-cell lung cancer. N. Engl. J. Med..

[8] Douillard J.-Y., Rosell R., Lena M.D., Carpagnano F., Ramlau R., Gonzáles-Larriba J.L., Grodzki T., Pereira J.R., Groumellec A.L., Lorusso V. (2006). Adjuvant vinorelbine plus cisplatin versus observation in patients with completely resected stage IB–IIIA non-small-cell lung cancer (Adjuvant Navelbine International Trialist Association [ANITA]): A randomised controlled trial. Lancet Oncol..

[9] Felip E., Altorki N., Zhou C., Csőszi T., Vynnychenko I., Goloborodko O., Luft A., Akopov A., Martinez-Marti A., Kenmotsu H. (2021). Adjuvant atezolizumab after adjuvant chemotherapy in resected stage IB-IIIA non-small-cell lung cancer (IMpower010): A randomised, multicentre, open-label, phase 3 trial. Lancet.

[10] Non-Small Cell Lung Cancer—Guidelines Detail. https://www.nccn.org/guidelines/guidelines-detail?category=1&id=1450.

[11] Rosa K. Adjuvant Atezolizumab Approved in Europe for High-Risk NSCLC with PD-L1 of ≥50%|OncLive. https://www.onclive.com/view/adjuvant-atezolizumab-approved-in-europe-for-high-risk-nsclc-with-pd-l1-of-50-.

[12] O’Brien M., Paz-Ares L., Marreaud S., Dafni U., Oselin K., Havel L., Esteban E., Isla D., Martinez-Marti A., Faehling M. (2022). Pembrolizumab versus placebo as adjuvant therapy for completely resected stage IB–IIIA non-small-cell lung cancer (PEARLS/KEYNOTE-091): An interim analysis of a randomised, triple-blind, phase 3 trial. Lancet Oncol..

[13] Wu Y.-L., John T., Grohe C., Majem M., Goldman J.W., Kim S.-W., Kato T., Laktionov K., Vu H.V., Wang Z. (2022). Postoperative Chemotherapy Use and Outcomes From ADAURA: Osimertinib as Adjuvant Therapy for Resected EGFR-Mutated NSCLC. J. Thorac. Oncol..

[14] Zhong W.-Z., Wang Q., Mao W.-M., Xu S.-T., Wu L., Shen Y., Liu Y.-Y., Chen C., Cheng Y., Xu L. (2018). Gefitinib versus vinorelbine plus cisplatin as adjuvant treatment for stage II-IIIA (N1-N2) EGFR-mutant NSCLC (ADJUVANT/CTONG1104): A randomised, open-label, phase 3 study. Lancet Oncol..

[15] Wu Y.-L., Dziadziuszko R., Ahn J.S., Barlesi F., Nishio M., Lee D.H., Lee J.-S., Zhong W., Horinouchi H., Mao W. (2024). Alectinib in Resected ALK-Positive Non–Small-Cell Lung Cancer. N. Engl. J. Med..

[16] Goss G., Darling G.E., Westeel V., Nakagawa K., Sureda B.M., Perrone F., McLachlan S.-A., Kang J.H., Wu Y.-L., Dingemans A.-M.C. (2024). LBA48 CCTG BR.31: A global, double-blind placebo-controlled, randomized phase III study of adjuvant durvalumab in completely resected non-small cell lung cancer (NSCLC). Ann. Oncol..

[17] National Cancer Institute (NCI) (2025). Adjuvant Nivolumab in Resected Lung Cancers (ANVIL)-A Randomized Phase III Study of Nivolumab After Surgical Resection and Adjuvant Chemotherapy in Non-Small Cell Lung Cancers. ClinicalTrials.gov. https://clinicaltrials.gov/study/NCT02595944.

[18] Sands J.M., Mandrekar S.J., Kozono D., Oxnard G.R., Hillman S.L., Wigle D.A., Govindan R., Carlisle J., Gray J., Salama J.K. (2021). Integration of immunotherapy into adjuvant therapy for resected non-small-cell lung cancer: ALCHEMIST chemo-IO (ACCIO). Immunotherapy.

[19] Chaft J.E., Oezkan F., Kris M.G., Bunn P.A., Wistuba I.I., Kwiatkowski D.J., Owen D.H., Tang Y., Johnson B.E., Lee J.M. (2022). Neoadjuvant atezolizumab for resectable non-small cell lung cancer: An open-label, single-arm phase II trial. Nat. Med..

[20] Forde P.M., Chaft J.E., Smith K.N., Anagnostou V., Cottrell T.R., Hellmann M.D., Zahurak M., Yang S.C., Jones D.R., Broderick S. (2018). Neoadjuvant PD-1 Blockade in Resectable Lung Cancer. N. Engl. J. Med..

[21] Cascone T., William W.N., Weissferdt A., Leung C.H., Lin H.Y., Pataer A., Godoy M.C.B., Carter B.W., Federico L., Reuben A. (2021). Neoadjuvant nivolumab or nivolumab plus ipilimumab in operable non-small cell lung cancer: The phase 2 randomized NEOSTAR trial. Nat. Med..

[22] Shu C.A., Grigg C., Chiuzan C., Garofano R.F., Patel V., Hernandez S., Negri T., Sacher A.G., Smith-Marrone S., Stoopler M. (2018). Neoadjuvant atezolizumab + chemotherapy in resectable non-small cell lung cancer (NSCLC). J. Clin. Oncol..

[23] Forde P.M., Spicer J., Lu S., Provencio M., Mitsudomi T., Awad M.M., Felip E., Broderick S.R., Brahmer J.R., Swanson S.J. (2022). Neoadjuvant Nivolumab plus Chemotherapy in Resectable Lung Cancer. N. Engl. J. Med..

[24] Spicer J., Girard N., Provencio M., Wang C., Mitsudomi T., Awad M.M., Vokes E.E., Taube J.M., Lupinacci L., Saylors G.B. (2024). Neoadjuvant nivolumab (NIVO) + chemotherapy (chemo) vs chemo in patients (pts) with resectable NSCLC: 4-year update from CheckMate 816. J. Clin. Oncol..

[25] Provencio M., Nadal E., González-Larriba J.L., Martínez-Martí A., Bernabé R., Bosch-Barrera J., Casal-Rubio J., Calvo V., Insa A., Ponce S. (2023). Perioperative Nivolumab and Chemotherapy in Stage III Non–Small-Cell Lung Cancer. N. Engl. J. Med..

[26] Provencio M., Nadal E., Insa A., Campelo R.G., Casal J., Dómine M., Massuti B., Majem M., Rodríguez-Abreu D., Martínez-Martí A. (2024). Perioperative chemotherapy and nivolumab in non-small-cell lung cancer (NADIM): 5-year clinical outcomes from a multicentre, single-arm, phase 2 trial. Lancet Oncol..

[27] Cascone T., Awad M.M., Spicer J.D., He J., Lu S., Sepesi B., Tanaka F., Taube J.M., Cornelissen R., Havel L. (2024). Perioperative Nivolumab in Resectable Lung Cancer. N. Engl. J. Med..

[28] Spicer J.D., Gao S., Liberman M., Kato T., Tsuboi M., Lee S.-H., Chen K.-N., Dooms C., Majem M., Eigendorff E. (2023). LBA56 Overall survival in the KEYNOTE-671 study of perioperative pembrolizumab for early-stage non-small-cell lung cancer (NSCLC). Ann. Oncol..

[29] Rothschild S.I., Zippelius A., Eboulet E.I., Savic Prince S., Betticher D., Bettini A., Früh M., Joerger M., Lardinois D., Gelpke H. (2021). SAKK 16/14: Durvalumab in Addition to Neoadjuvant Chemotherapy in Patients with Stage IIIA(N2) Non-Small-Cell Lung Cancer-A Multicenter Single-Arm Phase II Trial. J. Clin. Oncol..

[30] Heymach J.V., Harpole D., Mitsudomi T., Taube J.M., Galffy G., Hochmair M., Winder T., Zukov R., Garbaos G., Gao S. (2023). Perioperative Durvalumab for Resectable Non–Small-Cell Lung Cancer. N. Engl. J. Med..

[31] Toripalimab Plus Chemotherapy in Neoadjuvant/Adjuvant Settings Improves EFS vs Chemotherapy Alone in Stage III NSCLC. https://dailynews.ascopubs.org/do/10.1200/ADN.23.201345/full/.

[32] AstraZeneca (2025). A Phase II, Open-label, Multicentre, Randomised Study of Neoadjuvant and Adjuvant Treatment in Patients with Resectable, Early-Stage (II to IIIB) Non-Small Cell Lung Cancer (NeoCOAST-2). ClinicalTrials.gov. https://clinicaltrials.gov/study/NCT05061550.

[33] Novartis Pharmaceuticals (2025). Phase II Trial of Neoadjuvant and Adjuvant Capmatinib in Participants with Stages IB-IIIA, N2 and Selected IIIB (T3N2 or T4N2) NSCLC with MET Exon 14 Skipping Mutation or High MET Amplification (Geometry-N). ClinicalTrials.gov. https://clinicaltrials.gov/study/NCT04926831.

[34] Eli Lilly and Company (2025). LIBRETTO-432: A Placebo-Controlled Double-Blinded Randomized Phase 3 Study of Adjuvant Selpercatinib Following Definitive Locoregional Treatment in Participants with Stage IB-IIIA RET Fusion-Positive NSCLC. ClinicalTrials.gov. https://clinicaltrials.gov/study/NCT04819100.

[35] Genentech, Inc. (2025). NAUTIKA1: Multicenter, Phase II, Neoadjuvant and Adjuvant Study of Multiple Therapies in Biomarker-Selected Patients with Resectable Stages IB-III Non-Small Cell Lung Cancer. ClinicalTrials.gov. https://clinicaltrials.gov/study/NCT04302025.

[36] Li M.S. (2025). Neoadjuvant Sacituzumab Govitecan Plus Pembrolizumab in Resectable Non-Small Cell Lung Cancer: An Open-Label, Multicenter, Single Arm Phase 2 Study. ClinicalTrials.gov. https://clinicaltrials.gov/study/NCT06055465.

[37] Jones D.R., Opitz I., Harpole D., Yanagawa J., Lim E., Tsutani Y., Tan D.S.W., Dacic S., Ganti A.K., Bodla S. (2026). TROPION-Lung12: A phase 3 study of adjuvant datopotamab deruxtecan and rilvegostomig in ctDNA-positive or high-risk pathology stage I non-small cell lung cancer. J. Thorac. Cardiovasc. Surg..

[38] Cascone T., Bonanno L., Guisier F., Insa A., Liberman M., Bylicki O., Livi L., Egenod T., Corre R., Kim D.-W. (2025). Perioperative durvalumab plus chemotherapy plus new agents for resectable non-small-cell lung cancer: The platform phase 2 NeoCOAST-2 trial. Nat. Med..

[39] Memorial Sloan Kettering Cancer Center (2026). A Phase II Trial of Neoadjuvant Trastuzumab Deruxtecan for Patients with Stage II-III HER2-Amplified or HER2-Mutated Non-Small Cell Lung Cancer (HERCULES). ClinicalTrials.gov. https://clinicaltrials.gov/study/NCT07428044.

[40] Khadela A., Postwala H., Rana D., Dave H., Ranch K., Boddu S.H.S. (2023). A review of recent advances in the novel therapeutic targets and immunotherapy for lung cancer. Med. Oncol..

[41] Khadela A., Chavda V.P., Postwala H., Ephraim R., Apostolopoulos V., Shah Y. (2023). Configuring Therapeutic Aspects of Immune Checkpoints in Lung Cancer. Cancers.

[42] Liu J., Blake S.J., Yong M.C.R., Harjunpää H., Ngiow S.F., Takeda K., Young A., O’Donnell J.S., Allen S., Smyth M.J. (2016). Improved Efficacy of Neoadjuvant Compared to Adjuvant Immunotherapy to Eradicate Metastatic Disease. Cancer Discov..

[43] Ahern E., Solomon B.J., Hui R., Pavlakis N., O’Byrne K., Hughes B.G.M. (2021). Neoadjuvant immunotherapy for non-small cell lung cancer: Right drugs, right patient, right time?. J. Immunother. Cancer.

[44] Kalvapudi S., Vedire Y., Yendamuri S., Barbi J. (2023). Neoadjuvant therapy in non-small cell lung cancer: Basis, promise, and challenges. Front. Oncol..

[45] Gulack B.C., Cox M.L., Yang C.-F.J., Speicher P.J., Kara H.V., D’Amico T.A., Berry M.F., Hartwig M.G. (2018). Survival after radiation for stage I and II non-small cell lung cancer with positive margins. J. Surg. Res..

[46] Rajaram R., Huang Q., Li R.Z., Chandran U., Zhang Y., Amos T.B., Wright G.W.J., Ferko N.C., Kalsekar I. (2024). Recurrence-Free Survival in Patients with Surgically Resected Non-Small Cell Lung Cancer: A Systematic Literature Review and Meta-Analysis. CHEST.

[47] Choi P.J., Jeong S.S., Yoon S.S. (2013). Prognosis of Recurrence after Complete Resection in Early-Stage Non-Small Cell Lung Cancer. Korean J. Thorac. Cardiovasc. Surg..

[48] Son J.W., Lee J., Jeon J.H., Cho S., Jung W., Shih B.C.-H., Kim K., Jheon S. (2024). Validation of IASLC 9th edition TNM classification for lung cancer: Focus on N descriptor. BMC Cancer.

[49] Pignon J.-P., Tribodet H., Scagliotti G.V., Douillard J.-Y., Shepherd F.A., Stephens R.J., Dunant A., Torri V., Rosell R., Seymour L. (2008). Lung Adjuvant Cisplatin Evaluation: A Pooled Analysis by the LACE Collaborative Group. J. Clin. Oncol..

[50] Lisberg A., Cummings A., Goldman J.W., Bornazyan K., Reese N., Wang T., Coluzzi P., Ledezma B., Mendenhall M., Hunt J. (2018). A Phase II Study of Pembrolizumab in EGFR-Mutant, PD-L1+, Tyrosine Kinase Inhibitor Naïve Patients with Advanced NSCLC. J. Thorac. Oncol..

[51] Hsu K.-H., Tseng J.-S., Yang T.-Y., Chen K.-C., Su K.-Y., Yu S.-L., Chen J.J.W., Huang Y.-H., Chang G.-C. (2022). PD-L1 strong expressions affect the clinical outcomes of osimertinib in treatment naïve advanced EGFR-mutant non-small cell lung cancer patients. Sci. Rep..

[52] Kumar A., Tohmasi S., Eaton D.B., Seyoum N., Brandt W.S., Thomas T.S., Schoen M.W., Rossetti N.E., Chang S.-H., Yan Y. (2025). Real-World Adoption of Adjuvant Therapies for Resected Stage IB–III Non-Small-Cell Lung Cancer. Cancers.

[53] Guan S., Wang H., Chen Z., Guo F., Yi G., Du X., Yan J., Tian C. (2025). Is adjuvant immunotherapy necessary after neoadjuvant chemoimmunotherapy in patients with resectable stage III NSCLC? A two-center real-world study. Cancer Immunol. Immunother..

[54] Mao Y., Chen F., Ye Z., Li Z., Fan B., Zou Y., Li W., Lan F. (2024). Neoadjuvant immune checkpoint inhibitor reduced recurrence in operable NSCLC patients with pathological complete response: A retrospective analysis. BMC Cancer.

[55] Li H., Pezeshkian F., Xie Y., Mazzola E., Barcelos R.R., Hooshmand F., Han T., Laaksonen S., Sholl L., Vega C. (2025). Lung cancer recurrence after neoadjuvant immunotherapy. J. Thorac. Cardiovasc. Surg..

[56] Ohara S., Suda K., Tsutani Y. (2025). Utility and Future Perspectives of Circulating Tumor DNA Analysis in Non-Small Cell Lung Cancer Patients in the Era of Perioperative Chemo-Immunotherapy. Cells.

[57] Ferreira P.M., Campos R., Valente C., Ferreira J., Freitas C., Sousa C., Araújo D., Bastos H.N., Magalhães A., Fernandes M.G.O. (2025). Out with the old—Advancements and shortcomings of the updated 9th edition of the Tumor, Node, Metastasis (TNM) classification system for lung cancer. J. Bras. Pneumol..

[58] Helwick C. Patient-Level Data Support Perioperative Use of Nivolumab in Resectable NSCLC. https://ascopost.com/issues/november-25-2024-supplement-conference-highlights-2024-wclc/patient-level-data-support-perioperative-use-of-nivolumab-in-resectable-nsclc/.

